# Mechanical Dilation of the Recipient Vessel with the DeBakey Vascular Dilator in Lower Extremity Reconstruction: A Report of Two Cases

**DOI:** 10.1055/s-0043-1764309

**Published:** 2023-05-29

**Authors:** Min-Gi Seo, Tae-Gon Kim

**Affiliations:** 1 Department of Plastic and Reconstructive Surgery, College of Medicine, Yeungnam University, Daegu, Korea

**Keywords:** DeBakey vascular dilator, recipient vessel, lower extremity reconstruction, vasospasm, atherosclerosis

## Abstract

In lower extremity reconstruction, the recipient vessel often requires long-range mechanical dilation because of extensive vasospasm or plaque formation induced by concomitant atherosclerosis. While a forceps dilator can be used to manipulate and dilate vessels approximately 1 cm from their end, a DeBakey vascular dilator can dilate long-range vessels. The authors successfully performed free flap reconstruction of the lower extremity using the DeBakey vascular dilator. Of the two patients who underwent lower extremity reconstruction, one had extensive vasospasm, and the other had plaques in the recipient arteries. Irrigation with 4% lidocaine and dilation of the lumen with a forceps dilator were insufficient to restore the normal arterial blood flow. Instead, a DeBakey vascular dilator with a 1-mm diameter tip was gently inserted into the lumen. Then, to overcome vessel resistance, the dilator gently advanced approximately 10 cm to dilate the recipient artery. Normal arterial blood flow was gushed out after dilating the vessel lumen using a DeBakey vascular dilator. The vascular anastomosis was performed, and intravenous heparin 5000 IU was administered immediately after anastomosis. Prophylactic low-molecular-weight-heparin (Clexane, 1 mg/kg) was administered subcutaneously to both patients for 14 days. The reconstructed flap survived without necrosis in either patient.

## Introduction

The relatively low success rates of free flap surgeries in the lower extremity are due to the concomitant peripheral vascular disease, atherosclerosis, and vasospasm that are common in the lower extremity. 1 Therefore, the patient's preoperative vascular condition is usually evaluated, and percutaneous transluminal angioplasty is also performed if necessary to overcome these challenges. 2

Despite preoperative preparations for these risk factors, microsurgeons occasionally encounter insufficient blood flow in the recipient vessel of the lower extremity during surgery. Consequently, vessel manipulations such as intraluminal dilation of the recipient vessel, administration of topical vasodilator, and heparinized saline infusion may be performed to restore blood flow. 3 4 The forceps vessel dilator is commonly used to dilate the short-range lumen of the vessel from the vessel end, and blood flow is usually restored using this short-range intraluminal dilation. However, long-range mechanical dilation is required if there are extensive vasospasms or atherosclerosis in the recipient vessel. Therefore, the authors would like to introduce the experiences for restoring blood flow in the recipient vessel with dilation of the long-range lumen with the DeBakey vascular dilator and performing successful free flap surgeries in the lower extremity. The patients' consent was obtained for their clinical photographs for academic purposes. This study was conducted in accordance with the Declaration of Helsinki (as revised in 2013) and approved by the Institutional Review Board of Yeungnam University Hospital (No. 2022-09-041: the registration number of the ethics board).

## Case 1



**Video 1** The recipient vessel was first dilated using a forceps dilator. However, this was insufficient to restore normal arterial blood flow.


**Video 2** A DeBakey vascular dilator was inserted and gently advanced into the recipient vessel. Normal arterial blood flow was gushed out after long-range dilating of the vessel lumen using the DeBakey vascular dilator.


A 38-year-old male patient without any underlying disease was scheduled to undergo extensive resection for squamous cell carcinoma of the distal margin of the stump of the right foot. Preoperative Doppler ultrasound confirmed that the pulse and blood flow of the right anterior tibial artery (ATA), which was to be used as the recipient vessel, was intact. After extensive resection, the right anterolateral thigh (ALT) flap containing a 7 × 7 cm ^2^ -sized skin paddle was harvested to cover the defect. The right ATA was cut for microvascular anastomosis and normal arterial blood flow was confirmed. However, the blood flow weakened after a while because of vasospasms. Irrigation with 4% lidocaine and heparinized saline infusion was repeated at the end of the recipient vessel. Although the recipient vessel end was manipulated using a forceps dilator, normal blood flow was still not restored ( Video 1 ).

**Fig. 1.**
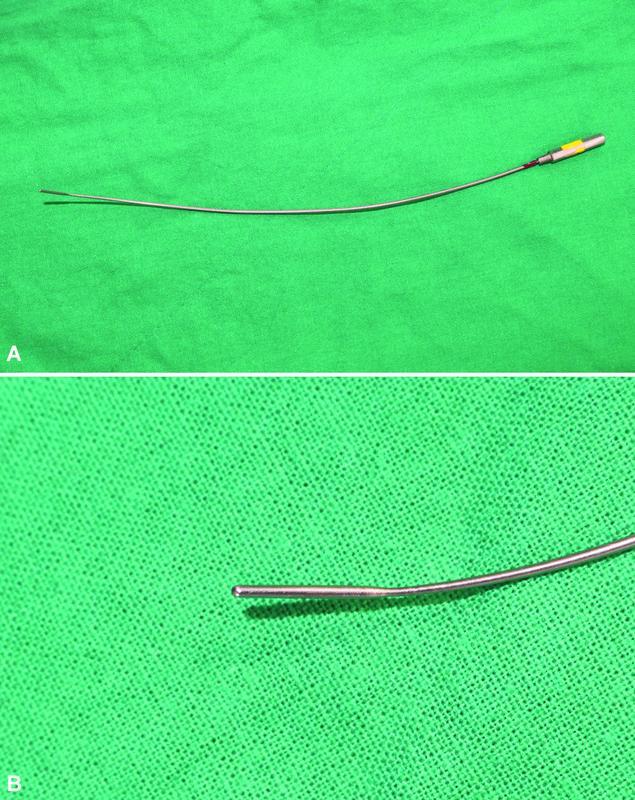
( **A** ) DeBakey vascular dilator (Garrett Nitinol Vascular Dilator). ( **B** ) A 1-mm diameter tip to dilate the vessels.

Consequently, a DeBakey vascular dilator with a 1-mm diameter tip was inserted into the recipient vessel lumen ( Fig. 1 ). The DeBakey vascular dilator was advanced gently and slowly, with caution to avoid delamination or intimal endothelial injury. Gently overcoming the resistance of the narrow lumen, it advanced by approximately 10 cm and extended the right ATA. The advance and retreat of the DeBakey vascular dilator were repeated several times until normal arterial blood flow was restored. After dilation, the arterial blood flow gushed out from the right ATA was confirmed ( Video 2 ). The distal end of the right ATA was partially resected, and end-to-end microvascular anastomosis was performed. Intravenous heparin 5000 IU was administered immediately after anastomosis and prophylactic low-molecular-weight-heparin (Clexane, 1 mg per kg) was subcutaneously injected for 14 days postoperatively. The patient did not have postoperative complications such as distal flap necrosis.

## Case 2

A 58-year-old male patient with hypertension and a cerebrovascular accident was scheduled to undergo extensive resection for malignant melanoma of the left heel. Preoperative three-dimensional angiography computed tomography revealed that atherosclerotic changes accompanied the lower extremity vessels. However, the patient did not undergo vascular interventions, such as percutaneous transluminal angioplasty, in advance. After extensive resection, the left ALT flap containing a 7 × 9 cm ^2^ -sized skin paddle was harvested to cover the defect. The side branch of the left posterior tibial artery (PTA) was cut for use as the recipient vessel during surgery. Weak blood flow in the recipient vessel was immediately confirmed owing to the plaque formation inside the lumen. Next, 4% lidocaine irrigation, heparinized saline infusion, and intraluminal dilation using a forceps dilator were conducted. However, normal arterial blood flow still was not gushed out. Then, a DeBakey vascular dilator with a 1-mm diameter tip was gently inserted into the lumen of the side branch of the left PTA and advanced enough to reach the main branch of the left PTA. After this, normal arterial blood flow was gushed out through long-range dilation of the recipient vessel, and end-to-end microvascular anastomosis was performed. Heparin 5000 IU was administered intravenously immediately after the anastomosis, and prophylactic low-molecular-weight-heparin was administered for 14 days. There were no postoperative complications such as necrosis of the distal flap.

## Discussion

DeBakey vascular dilators are commonly used during cardiovascular surgeries to confirm and dilate coronary artery stenosis or dilate the lumen of the harvested internal mammary artery (IMA) to resolve arterial vasospasms. 5 The forceps dilator is mainly used for vessel dilation in microsurgery, while the DeBakey vascular dilator is rarely used. A case in which the IMA was dilated using a DeBakey vascular dilator in autologous breast reconstruction has been reported. 6 However, there have been no case reports on using the DeBakey vascular dilator during free flap surgeries of the lower extremity.

The choice of the recipient vessel in lower extremity reconstruction is limited, and concomitant atherosclerosis or diabetes mellitus makes the selection particularly difficult due to the narrowing vessel lumen. 1 Even if a recipient vessel is selected through proper preoperative preparation, unexpected vasospasm may interrupt microvascular anastomosis. Vasospasm of arteries can result from the contraction of the smooth muscles in the arterial media following trauma, cold temperature, and traction on the vessel wall. 7 8 Vasospasm of recipient vessels can increase failure rates in free flap surgeries, especially in posttraumatic perivascular-injured vessels. 9 And vessels in the lower extremity are more susceptible to vasospasm. 7 Therefore, it is important to properly manipulate the recipient vessel with atherosclerosis or vasospasm during the lower extremity reconstruction.

Normal blood flow can usually be restored by dilating the short-range lumen of the vessel with a forceps dilator. 8 However, if normal blood flow is not restored after using the forceps dilator, the long-range lumen of the recipient vessel might be narrowed. In this situation, the long-range recipient vessel can be manipulated and dilated using a DeBakey vascular dilator. A DeBakey vascular dilator with a streamlined tip with a diameter equal to or smaller than the recipient vascular diameter should be selected to minimize injury to the recipient vessel, and a step-by-step use of the dilator from small caliber to large caliber is required. 4 Excessive vessel traction and grasping should be avoided during manipulation. 9 The DeBakey vascular dilator must be inserted into the lumen very gently with care to prevent vascular intimal injury. The advance and retreat of the DeBakey vascular dilator can be repeated several times until the normal blood flow of the recipient vessel is gushed out.

The intimal layer of atherosclerotic vessels is fragile and easily damaged due to vessel manipulation and suturing. 9 Even in the absence of atherosclerosis, vessel delamination and intimal injury can occur during mechanical vessel dilation. These vascular injuries can cause platelet aggregation, subsequent thrombus formation, and vasospasm. 7 In particular, the DeBakey vascular dilator may lead to extensive intimal injuries because it can be inserted into the long-range lumen. In addition to intraoperative heparin administration and careful vessel manipulation, prophylactic use of low-molecular-weight-heparin after surgery can help prevent thrombus formation owing to possible intimal injury. 10 11 12 13

In free flap surgeries of the lower extremity, conventional manipulation with a forceps dilator alone is occasionally insufficient to restore the blood flow of the recipient vessel. Hence, if mechanical dilation of a long-range recipient vessel is required, a DeBakey vascular dilator can be used to overcome this limitation.
